# Community of Inquiry framework to evaluate an online obstetric and neonatal emergency simulation workshop for health professional students in India

**DOI:** 10.1186/s41077-022-00220-5

**Published:** 2022-08-24

**Authors:** Nisha Khot, Mahbub Sarkar, Utkarsh Bansal, Jai Vir Singh, Pramod Pharande, Atul Malhotra, Arunaz Kumar

**Affiliations:** 1 Royal Women’s Hospital, Melbourne, Australia; 2 Monash Centre for Scholarship in Health Education, Monash University, Melbourne, Australia; 3 Hind Institute of Medical Sciences, Barabanki, Uttar Pradesh India; 4 Monash Newborn, Monash Children’s Hospital, Melbourne, Australia; 5 Department of Paediatrics, Monash University, 246 Clayton Road, Clayton, Melbourne, VIC 3168 Australia; 6 Department of Obstetrics and Gynaecology, Monash University, Melbourne, Australia

**Keywords:** Education, Emergency, Interprofessional, Learning, ONE-Sim

## Abstract

**Background:**

We transitioned our obstetric neonatal emergency simulation (ONE-Sim) workshops to an online format during the COVID-19 pandemic. In this study, we evaluated key learning acquired by undergraduate medical and nursing students attending the online ONE-Sim workshops from a low- and middle-income country (LMIC).

**Methods:**

Student perception of online workshops was collected using electronic questionnaires. Data was analysed using thematic analysis by employing the Community of Inquiry (CoI) framework.

**Results:**

One hundred sixty medical and nursing students who attended the online ONE-Sim workshops completed the questionnaires. There was evidence in the data to support all three aspects of the CoI framework—social, cognitive and teacher presence.

**Conclusions:**

The use of the CoI framework helped to describe key learning from online interprofessional simulation workshops conducted for a LMIC.

## Introduction

Simulation-based education (SBE) is a well-established component of health professional education. While SBE does not replace clinical workplace-based learning, it is an important adjunct to training and provides students with “scaffold” learning to develop clinical skills [34]. SBE for undergraduate medical and nursing education is relatively new in low-middle income countries (LMICs) [36] as is inter-professional education (IPE). Inter-professional SBE is particularly relevant in LMICs like India with high rates of maternal and perinatal mortality [20].

Introduction of IPE early during undergraduate training can increase student’s exposure to other professions and allow them to develop unprejudiced impressions before they graduate [29]. IPE has been shown to improve cooperation and reduce errors arising from miscommunication [50] during perinatal emergencies. The obstetric neonatal emergency simulation (ONE-Sim) programme is one of the few simulation-based interprofessional training programmes for undergraduate medical and nursing students in India [27].

The impact that COVID-19 restrictions placed on conducting in-person team training led to a transition to online teaching and learning for all learners including medical, nursing and midwifery students [38, 42, 51]. There are a variety of challenges to simulation-based team training specifically in low-resource settings that were compounded by the current pandemic: lack of funding, shortage of skilled educators, poor local infrastructure and limited health supplies. This creates a need for developing a sustainable educational approach, where online learning can assist in filling the learning gap to some extent. Strategies to maintain simulation-based team training in maternity care while complying with distancing regulations have been reported in countries where SBE is well embedded in healthcare training [24, 37]. There have also been reports from low-middle income countries of adaptations to enable ongoing clinical education during the pandemic [48]. These strategies include using online learning environments.

To create an engaging online learning experience, healthcare educators must learn important technologic advances, using media that are effective both interpersonally and academically [12, 16]. Additionally, explicit frameworks are critical for conceptual transferability of programmes offering useful models to other educators [46]. Despite the plethora of research on converting face-to-face educational activities into virtual platforms, few have described conceptual frameworks that underpin their application and development [21]. To maintain high-quality learning from online SBE, it is useful to implement conceptual frameworks but there is a paucity of literature describing the use of frameworks such as the Community of Inquiry (CoI) framework [10, 19, 31]. In our study, we describe the use of the Community of Inquiry (CoI) framework [19] to guide the design, delivery, and evaluation of the online ONE-Sim workshop.

### Community of Inquiry (CoI) framework

Higher education has consistently viewed community as essential to support collaborative learning and discourse associated with higher learning. Although online learning communities can be disconnected, there is evidence that a community of learners can be created online [41, 49]. The Community of Inquiry (CoI) model [17] provides a collaborative-constructivist theoretical framework to understand the dynamics of an online learning experience. The CoI has three interdependent elements essential to educational transactions—*cognitive presence*, *social presence and teaching presence*.

Garrison et al. operationalised cognitive presence in a four-phase process: triggering event, exploration, integration and application [18]. As such, it reflects the purposeful nature of collaborative knowledge construction.

The second component, social presence, is defined as the ability of participants to identify with the community (in this context, the simulation workshop), communicate purposefully in a trusting environment and develop inter-personal relationships (in this case with midwifery and medical students) by way of projecting their individual personalities [22]. The three categories of open communication, group cohesion and personal projection are used to operationalise this concept.

The third component, teaching presence, consists of three functions of teacher responsibility—design of the teaching experience, facilitation and direct instruction [2]. The first function engages teachers to select, organise and present course content, along with designing and developing learning activities and assessment. The second function is shared among teachers and participants. The third function is important in a formal educational context, where there will be times when it is necessary to intervene directly. Each of these functions is associated with integration of social and cognitive processes to make the learning experience purposeful.

CoI has been used in synchronous online learning in healthcare [43]. It was considered as an appropriate framework to evaluate this research, as this was an online education involving multiple players from different health professions, co-delivered by faculty located overseas. All three components of CoI were thought to be relevant for the programme delivery. There is, however, limited knowledge of how CoI can inform the online simulation-based education in the healthcare setting. Using the CoI framework, the current study was designed, where the key research question was to explore the participants’ perceptions of the impact of the online ONE-Sim programme.

## Methods

### Study design

Drawing on CoI [19], the current study employed qualitative methodology to explore medical and midwifery students’ perceptions of the impact of online simulation programme—ONE-Sim. This study particularly employed a qualitative descriptive design to present a comprehensive descriptive summary of participants’ perceived impact of the ONE-Sim programme on their preparedness of practice, without abstract rendering of data [14]. Although the CoI framework was not taught to the participants, nor was it used to guide the teaching during the workshop, it was used mainly as the lens to guide the evaluation of the online ONE-Sim programme. The study obtained ethics approval from Hind Institute of Medical Sciences, Uttar Pradesh, India.

### ONE-Sim programme

The ONE-Sim is an interprofessional SBE programme for health professionals and students involved in childbirth, where participants manage obstetric and neonatal emergency scenarios as a team. We have previously described the ONE-Sim hands-on workshops conducted using blended simulation [27, 52]. The focus of the programme is to provide hands-on skills training to the participants in dealing with perinatal emergencies and management of these emergencies using a team-based learning approach. This is complemented by in-depth discussions (debrief) about teamwork and behavioural aspects of crisis resource management.

#### Setting and equipment for online ONE-Sim

The current study describes the online format of the ONE-Sim workshop where trained medical and midwifery facilitators demonstrated the clinical management through role-play [32]. The workshop including the emergency scenarios were live streamed from a high-technology simulation centre in Melbourne, Australia, to the learners’ homes in Uttar Pradesh, India. Co-authors AK and AM led the programme as they had academic affiliations at both Monash University, Melbourne, and Hind Institute of Medical Sciences, Lucknow (HIMS), India. They were assisted by other faculty members in Melbourne (NK, PP) and from HIMS (UK, JVS who were present online). A strategy was devised to make the workshops compliant with distancing requirements and adapted to an online format, with students learning online as observers of simulated emergencies.

The workshops started with initial briefing and introductions, laying the ground rules (confidentiality, physical and psychological safety), clarifying the learning objectives and familiarisation of the participants with equipment and simulation manikins. With hybrid simulation, medical and midwifery facilitators played the role of the participants in the scenario (including that of the standardised patient and the simulated woman responded to verbal prompts), while intimate examinations were performed using the childbirth simulator (Prompt Flex, Limbs and Things, Kent, UK). The scenario involving newborn resuscitation used a Newborn Anne model (Laerdal, Stavenger, Norway). A hand-held wide-angle camera device with a gimbal stabiliser was used for optimum viewing experience [32]. All facilitators wore appropriate personal protective equipment (PPE) and donning and doffing of PPE was demonstrated at the start of the workshop.

#### Simulated scenarios

The workshops covered two distinct clinical scenarios. Each scenario commenced with donning of PPE by the participants in a time-critical manner. Each scenario ended with doffing of PPE by the participants with attention to minimising self-contamination during the process. In the first scenario, an uncomplicated, normal, vaginal birth was demonstrated. The second scenario involved a birth complicated by obstructed labour (shoulder dystocia), with the baby requiring resuscitation, and subsequently postpartum haemorrhage in the mother.

#### Online debriefing session

Following the scenarios, small groups of participants (15–20 per group) were invited to online breakout rooms for debrief, using trigger question discussions to consolidate key learning. Each small group was facilitated by a minimum of 2 facilitators (an obstetrician, midwife or paediatrician) with prior experience in debriefing.

At the start of debrief, the goals and process for debriefing were made explicit. Learners were familiarised with the online environment like using the mute function to minimise disruption and displaying participant’s first names on their online profile to assist the facilitators to address learners by name. A gallery/grid view function was used so that facilitators could respond to non-verbal cues. Learners were also encouraged to use the grid view. The facilitators attempted to promote inclusivity by use of collective pronouns to refer to the group (e.g., Let us talk about what we observed during the resuscitation of the baby).

Each workshop lasted around 2 h (initial briefing 30 min, scenarios 30 min and debriefing 40–50 min with concluding messages discussed for 10 min).

### Participants

Participants included local medical (year 5 of a 5-year training course) and nursing/midwifery (year 4 of a 4-year training course) students. Participants were in their clinical years at a secondary level (metropolitan) teaching hospital in Uttar Pradesh, India. They attended one of the two online ONE-Sim workshops held at Hind Institute of Medical Sciences, Barabanki, Uttar Pradesh. All students were offered the opportunity to attend the workshop but attendance was entirely voluntary. Completion of the post-workshop questionnaire was also voluntary.

### Data collection

Data were collected using a post-workshop questionnaire administered online. The research team developed the questionnaire. Participants spent about 15–20 min completing it. The questionnaire started with asking participants to describe their professional characteristics (e.g., What is your clinical role?). It then included five open-ended questions addressing learning from the workshop and participants’ experience of the digital format (e.g., What do you think of gaining experience via zoom videoconferencing?).

To avoid any risk of coercion, administrative staff (not part of the research team) at HIMS distributed the questionnaire. The questionnaire was circulated 1–2 weeks following the workshop attendance to capture thick descriptions of student perspectives, with the assumption that this would provide time for students to reflect on their learning.

### Data analysis

Data from nursing/midwifery participants and medical participants were analysed. Medical student’s quotes were allocated the letter M followed by a participant number (from 1 to 71) while nursing/midwifery student’s quotes were allocated the letter N followed by a participant number (from 1 to 89). Authors NK and AK analysed the data employing a thematic analysis approach [5]. The process started with authors reading data multiple times to ensure familiarity and to develop a deeper understanding of the data. The authors then independently coded the data using the principles of framework analysis [15, 40] with the CoI framework and generated themes. They met a few times to discuss their analyses and the different insights they brought to their interpretations of the data. Based on their discussions, they identified indicators for the presence of each of the CoI framework and the different categories as well as indicators within each presence. These were further refined through discussions with other co-authors, AM and MS.

## Results

### Participant characteristics

A total of 160 students who attended the two online workshops completed the questionnaires. Of these, 71 (44.4%) were medical students, while 89 (55.6%) were nursing/midwifery students. Both medical and nursing/midwifery students were in their clinical years of their undergraduate course. A total of 69 (43.1%) participants had been previously exposed to SBE using a digital platform, but none of them had experienced online interprofessional education (IPE). Participant feedback was overwhelmingly positive (88% of medical students and 92% of nursing/midwifery students) regarding the innovative use of digital technology and the opportunity to experience clinical and teamwork skills.

### Community of Inquiry framework (Fig. 1)

We present how participants of our online workshop recognised the components of the CoI framework with description of the indicators in each section (Tables 1, 2 and 3). There was evidence in the data to support the presence of all three components of the CoI framework. Cognitive presence was indicated by a triggering event, exploration, integration by exchange of information and resolution with connecting disparate ideas and applying new ideas. Social presence was indicated by open communication, group cohesion and affective expression while teaching or educator presence was indicated by design and organisation, facilitation of discourse and direct instruction.

**Fig. 1:**
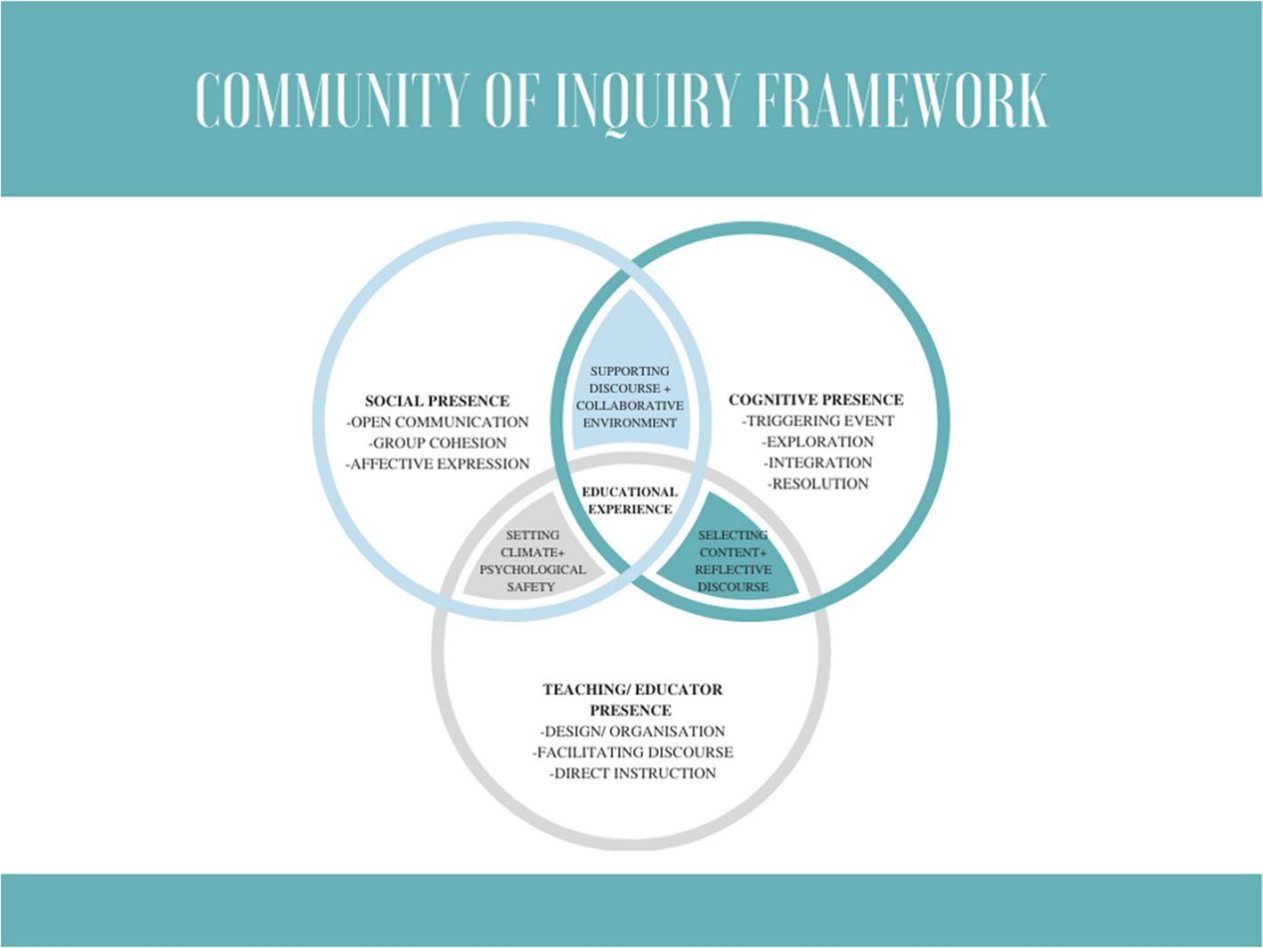
Community of Inquiry framework

**Table 1:** Categories, indicators and illustrative quotations for cognitive presence

Categories	Indicators	Example quotation
**Triggering event**	Novelty	1. “This was my first online workshop. I found this very new and interesting. Initially I felt that live is much more effective but at the end of the workshop, I found I had learned many new skills” M71
		2. “The online experience was very new, but it was a good way to gain experience” N34
	Sense of puzzlement	3. “I have not started clinical practice, so I don’t have much experience. This was my first time learning how to resuscitate a newborn. When the baby was delivered and needed resuscitation, I was looking for signals that the right procedure was being done.” N34
	Visualisation	4. “It felt like a real labour room” M28
		5. “The visual representation leaves a better memory and will help my future practice” N82
**Exploration**	Information exchange	6. “Interprofessional exchange of thoughts and information occurred on a common emergency situation in labour ward.” M30
	Learning different strategies	7. “The doctors and nurses checked repeatedly if the patient’s condition was improving. They used a wide range of problem-solving approaches to manage the condition” N24
	Establishing common goals	8. “Whether it is medical or nursing, we can’t achieve the goals without all team members knowledge of the goal” M41
		9. “It was helpful to see an ideal system where nurses and doctors all worked together towards a common goal” N27
	Using all available resources	10. “We have seen how to use whatever we have available in our hospital. And to get help from all staff” M26
		11. “I will check what resources I have wherever I work. This will help me manage complex situations better” N10
**Integration**	Connecting ideas	12. “We come to know about different solutions to the same problem and use a wide range of approaches. Multiple thoughts and queries can be exchanged which widens our minds to different ideas.” M21
	Team connection	13. “Teamwork is the key to any emergency. The team members worked collaboratively in the emergency” M32
		14. “The team worked together, did everything in the right sequence. They helped each other with the tasks” N51
		15. “Division of work is an important aspect while managing a patient. But the team has to stay connected while doing this” N30
	Connecting with the leader	16. “Every team member was completely aware of his job. They were willing to listen to the leader and respond to their requests” M47
		17. “The leader assigned the task, and everyone did their allocated task” N2
**Resolution**	Apply new ideas	18. “Only when we know what is normal can we recognise when things are not normal. After this workshop, I realised the importance of knowing normal first” M29
		19. “I learned a lot of new skills that I will be able to use in practice” N73
	Learning to re-evaluate	20. “When a particular approach did not work, I saw the team pause and review what they should do next” M32
		21. “The paediatric doctor checked baby’s heart rate and breathing many times. This helped him decide what to do next” N14
	Learner becomes the educator	22. “This will help me for future quality of patient care. It has improved my knowledge and I can use this to teach others.” M3
		23. “I am a student nurse now but when I am senior, I can use what I have learned today to teach my juniors” N87

**Table 2:** Categories, indicators and illustrative quotations for social presence

Categories	Indicators	Example quotation
**Open communication**	Risk free expression	1. “Doctors should communicate with nurses with respect.” M5
		2. “We are able to learn how to work with our colleagues, know that everyone is equally important despite their rank and to speak up even in front of seniors.” N11
	Flattening traditional hierarchical communication	3. “In some places there is no communication between nurses and doctors. Because of this patient outcomes suffer. It is good to conduct workshops together and learn how to share our experience with doctors” M51
		4. “An inter-professional workshop like this helps us to know the importance of each team member irrespective of qualification. Doctors and nurses work together and this helps everyone speak up despite their rank” N21
**Affective expression**	Emotions	5. “Performing procedures in an emergency in real life is scary” M52
		6. “I felt scared and panicky at the start” N1
	Staying calm	7. “I learned how to control my fear and stay calm on the outside” M13
		8. “Staying calm is an important leadership quality. When the leader is calm and confident, everyone else also feels confident” N21
	Communicating emotional response	9. “When there is an emergency, I get very nervous and make mistakes. I should let my team know this so that they can help me do my job better” M48
		10. “I have learned from this workshop how to communicate with my team when I am unsure of what to do” N44
**Group cohesion**	Encourage collaboration	11. “Everyone involved has a role to play and it is important to merge different ideas and cooperate with each other.” M33
		12. “In any emergency, it is important that skilled people from multiple professions work together to coordinate all the tasks.” N18
	Valuing collective effort over individual “heroism”	13. “Teamwork is important to achieve good outcomes. One person can’t do all the work at the same time” M27
		14. “Doctors, nurses and other staff make one big team. Without any one of them the structure would weaken as they are all pillars which uphold good patient care” N6
		15. “Saving a life is not a job anyone can do single-handedly” N33
	Team preparedness	16. “Planning and preparing before carrying out any procedure and appointing people best suited for specific tasks is the first priority” M10
		17. “Simulation is the best way I have learnt to recognise all team members are adequately prepared for all possible emergencies” N82
	Team culture/relationships	18. “This (workshop) will help me improve my interpersonal skills. This will help in forming trusting relationships” M4
		19. “Doing this workshop helps form a bond between medical and nursing staff. Trusting your teammates is important” N67

**Table 3:** Categories, indicators and illustrative quotations for teacher presence

Categories	Indicators	Example quotation
**Design and organisation**	Setting curriculum and methods	1. “The topics covered in the workshop are of great importance and the demonstration made every point clear as to how each step has to be taken” M53
		2. “The choice of topics was good because PPH is the most common emergency we see in practice” N18
	Stepped learning	3. “I found it easy to learn when every step was demonstrated clearly” M26
		4. “Any procedure can be learnt better if shown step by step…it helps students remember each step when performing the procedure in future” N82
	Developing familiarity via repetition	5. “I would attend another workshop because I may forget what was taught in a few months. Repeating the same workshop will help me remember” M39
		6. “Practicing the same scenario again and again helps us to manage an emergency in real-life easily” N73
**Facilitating discourse**	Creating opportunities for learning for all participants	7. “The teachers made sure everyone got a chance to speak up so every person could benefit” M6
		8. “The interaction with each other and sharing experiences meant that everyone got the opportunity to learn” N38
	Collective learning	9. “I believe it was very smart to include both medical and nursing students. This was we can get to know each other’s views and benefit from each other’s knowledge” M61
		10. “This (inter-professional learning) was the best part of the workshop. Nurses and doctors have a different approach. Learning together makes the combination of both skills a great experience” N26
**Direct instruction**	Assigning roles and responsibilities	11. “Division of work is an important aspect while managing the patient. Every person has a particular role to play. I saw the senior doctor become the leader and assign roles to each team member” M28
		12. “The work was equally distributed as per each team member’s skill. This made the critical situation easier to manage” N46
	Demonstrating technical skills	13. “Dr XYZ showed how to perform a normal birth and manage PPH. Each step and manoeuvre was clearly demonstrated” M18
		14. “Although it was difficult to see each manoeuvre in shoulder dystocia clearly, the person named each step so we could know what they were doing” N23
	Focusing direction	15. “At times, a person from a specific profession may be clueless when some in-depth discussion begins on a specific topic that they don’t know much about. This is when the teachers did an appreciable job to move the discussion back to the team.” M45
		16. “The instructions from the teachers gave clear direction to the students” N83

#### Cognitive presence

The online workshop as a triggering event was a novel experience for participants (Table 1, quotes 1–2). They expressed a sense of puzzlement (Table 1, quote 3) and highlighted the importance of visualisation (Table 1, quotes 4–5, “It felt like a real labour room”), and realism to enhance learning. Learners could establish reflective discourse by exchanging information (Table 1, quote 6) and learning to use different strategies (Table 1, quote 7) to identify and explore problems and establish common goals (Table 1, quotes 8–9, “We can’t achieve the goals without all team members knowledge of the goal”) using all available resources (Table 1, quotes 10–11, “I will check what resources I have to work with”). Learners could then progress to integration of the information to connect different ideas and thoughts (Table 1, quote 12). This led to establishing connections between team members (Table 1, quotes 13–15, “Teamwork is the key in any emergency”) as well as with the team leader (Table 1, quotes 16–17). The final step in cognitive presence of resolution occurred when participants could apply new ideas to the same situation (Table 1, quotes 18–19, “Only when we know what is normal can we recognise when things are not normal”). Thus, participants learned to re-evaluate and take collaborative responsibility for their own learning (Table 1, quotes 20–21). Finally, learners could see themselves as educators who would select content and guiding the pace and flow of discussion (Table 1, quotes 22-23, “I can use what I have learned today to teach my juniors”).

#### Social presence

Participants in the workshop communicated openly and felt that they could take interpersonal risks without fear of repercussions (Table 2, quotes 1–2). The ability to communicate openly was seen as a way of flattening the traditional hierarchical structure (Table 2, quotes 3–4, “An interprofessional workshop like this helps us know the importance of each team member irrespective of qualification”). Learners were able to protect their personal characteristics and identity while expressing their emotional responses to the evolving emergency (Table 2, quotes 5–6, “I felt scared and panicky at the start”). Participants recognised the importance of staying calm (Table 2, quotes 7–8) while also communicating vulnerability to the team in a safe way (Table 2, quotes 9–10). The ability to speak freely and manage emotions meant that the group was able to come together in a meaningful way. Cohesion within the groups encouraged collaboration (Table 2, quotes 11–12, “Everyone has a role to play”) and the group understood the value of collective effort rather than individual heroism (Table 2, quotes 13–15, “Saving a life is not a job anyone can do single-handedly”). The workshop was seen as a way of preparing for an emergency as a team (Table 2, quotes 16–17) and for building a team culture of trust (Table 2, quotes 18–19, “Trusting your teammates is important”).

#### Teacher/educator presence

Participants recognised various components of the design and organisation of the workshop. They found that the curriculum was appropriate and the demonstration method used was clear (Table 3, quotes 1–2). Learners also saw the value in stepped learning (Table 3, quotes 3–4, “I found it easy to learn when every step was demonstrated clearly”) and in developing familiarity with different emergency settings via repetition (Table 3, quotes 5–6).

Facilitating discourse is crucial to achieving learning objectives. The facilitators were able to create opportunities for learning for all participants (Table 3, quotes 7–8, “The teacher made sure everyone got a chance to speak”), drawing out those who were initially quiet and allowing equal time for all participants to speak. Both medical and nursing/midwifery students recognised the value of facilitated discussion by the educators between the professions (Table 3, quotes 9–10, “This i.e. interprofessional learning was the best part of the workshop”).

Direct instruction is an important component of the workshop. This includes assigning roles and responsibilities in a clear, unambiguous way (Table 3, quotes 11–12), demonstrating technical skills like resuscitation techniques (Table 3, quotes 13–14) and focussing on the timeline and direction of the clinical scenario (Table 3, quotes 15–16, “The instructions from teachers gave clear direction to the students”).

## Discussion

Online simulation programmes using web-based video-conferencing platforms have increased over the last few years [13, 33] but these necessary pedagogical adaptations have not been reported in the setting of perinatal emergency training in LMICs. Our study describes a novel online perinatal emergency simulation (ONE-Sim) workshop for students in India. The results show that undergraduate medical as well as nursing/midwifery students had a productive experience of SBE in an interprofessional learning environment. Our analysis highlights the use of the CoI framework to evaluate online simulation. The online workshop provided key messages of crisis resource management effective teamwork, leadership and communication. The online format may be a feasible alternative to in-person SBE while complying with COVID-related distancing requirements. It could also be used in other settings for example when training healthcare workers in remote locations and harder to reach communities.

Our study shows that participants of this novel online simulation workshop found it useful to develop teamwork skills especially at a time when clinical placements have been reduced or shortened. After watching facilitators simulate the emergency, students interacted with each other, both within and across professional boundaries to critically appraise teamwork, communication skills and collaboration. Both medical and nursing students recognised that the workshop promoted an approach of mutual respect for each team member. Participants learned the value of collective effort, particularly contextual in the Indian healthcare system where traditional hierarchical structures rely on doctors to make critical decisions and undervalue the contribution of nurses and midwives. A previous study of in-person IPE [27] reported on the divide between teams working in hierarchical work cultures and the role of IPE in flattening the hierarchy. The online workshop provided similar experience to the in-person workshops for learners across professional groups.

### Role of Community of Inquiry framework in health professional education

CoI has been studied extensively and literature supports its applicability to synchronous online learning in healthcare [43]. However, there are no reports of its use for online simulation-based education. A strong sense of community is essential to effective online learning environments. The three elements of CoI conceptualise how online learning spaces are jointly created by the manner in which educators plan and facilitate their session, how learners think and solve problems together and the ways in which all parties connect in an online context.

#### Cognitive presence

Sustained reflection and discourse leading to construction of meaning is the definition of the first component of CoI, namely, cognitive presence. It is operationalised in a four-phase process: triggering event, exploration, integration and resolution. The triggering event involves identification of a problem, exploration is where students explore the issue individually and collectively through critical reflection to allow integration, where learners construct meaning and resolution where the newly gained knowledge is applied to the workplace setting. Student groups comprised of different professions and a variety of personalities may be more effective in developing metacognitive interaction [30].

In our study, after watching facilitators simulate the emergency, students interacted with each other, both within and across professional boundaries to critically appraise teamwork, communication skills, collaboration and leadership. Using the evolving emergency as a triggering event, learners identified the critical role of a team leader. Using online breakout rooms, small group discussion between medical and nursing students identified the need for establishing leadership. Nursing students understood the need to make decisions independently initially until the arrival of a senior experienced practitioner when decision-making could be transferred appropriately. Medical students recognised the skills of nursing colleagues. They were able to appreciate nursing leadership in addition to effective medical leadership in an emergency.

It has been reported in literature that leadership is best established by the person who has the most experience of the emergency, who knows the team and is available to take the lead [6]. Although studies have identified leadership as pivotal in maintaining teamwork [3], there is little evidence to show how to establish leadership in the midst of clinical emergencies or how to teach leadership skills [45]. It is possible for medical and midwifery students to learn leadership skills and styles from their seniors by observing simulated scenarios followed by facilitated discussion [11].

#### Social presence

Of the three elements of CoI, the role of social presence has been studied most extensively. Research studies suggest a strong relationship between social presence and learning outcomes [25]. Social presence is the ability of learners and educators to project themselves socially and emotionally, thereby being perceived as “real people”. Collaborative activities allow learners greater sense of online community, which tends to support more rapid mastery of the “hidden curriculum”, in this case challenging traditional hierarchical structures and creating a positive work culture.

Three key components contribute to social presence, namely open communication, emotional/affective expression and group cohesion. Laying the ground rules provided learners with a safe space to take interpersonal risks without repercussions encouraged interprofessional collaboration and expression of emotions. Participants of the workshop learned the value of collective effort, particularly contextual in the Indian healthcare system, where traditional hierarchical structures rely on doctors to make critical decisions and undervalue the contribution of nurses and midwives. Demonstration of the use of different communication styles was seen as a way of flattening the hierarchy and promoting open discussion between doctors and nurses. Participants, on reflection, explicitly identified the experience of learning together as contributing to a positive work culture. A growing body of evidence suggests an association between positive workplace cultures and good patient outcomes [4]. Studies have also shown that interprofessional SBE is a useful intervention to improve workplace culture in maternity settings [28, 44]. Our study shows that similar improvement in workplace culture could be achieved in the Indian setting.

#### Teacher presence

In addition to facilitating discourse, the concept of educator or teaching presence, the third component of the CoI framework, includes two other aspects, namely instructional design and direct instruction. Instructional design is the deliberate creation of specific contextual scenarios within the workshop. Use of clear and consistent workshop structure with engaged instructors supports dynamic discussion, which in turn, is the most consistent predictor of successful online courses [7, 47]. During the debrief, the educator facilitates discussion to allow students to share meaning, identify areas of agreement as well as disagreement and seek to reach consensus. This includes creating opportunities for learning for all participants by drawing out inactive students and limiting the activity of dominant ones when they become detrimental to the learning of the group [1]. Strategies used to achieve this include visibility on video of both learners and educators, using the gallery or grid view and chatbox function and co-facilitating [8]. This is particularly important in perinatal emergency training where the emphasis is on developing team decision-making skills. The educator who is a subject matter expert (in this case an experienced obstetrician and paediatrician) is able to inject knowledge from reliable sources, correct misinterpretations and scaffold learner knowledge to raise it to a new level by using scripted phrases or conversational techniques to trigger reflection and discussion [39].

The workshop was designed to support this scaffolded learning allowing students to build on theoretical knowledge and progress to critical thinking and resolution. Participants in the workshop were able to clarify core knowledge and skills as well as build on this to visualise themselves as future teachers. Facilitated interaction between medical and nursing students led to recognition of the complementary nature of both professions. Creating effective learning scenarios is known to lead to better learner engagement. Appropriately designed scenarios can also increase the learner’s ability to communicate and care for patients [9]. Failing to recognise the collaborative and mutually dependant nature of medicine and midwifery could potentially compromise patient care [26]. Adopting similar learning outcomes for both medical and nursing/midwifery students may translate to fewer differences of opinion when they eventually become doctors and nurses/midwives [23].

In recent years, despite rapid transition from in-person to online SBE, there has been limited investigation of application of theoretical frameworks to online SBE. The CoI framework has the potential to address the intersection of pedagogy, technology and learners’ needs. The framework aligns well with the unique demands of online learning. Our study shows that the CoI framework could help formalise a structure for future workshops while providing an equivalent learning experience when compared with in-person SBE.

### Strengths and limitations

This is the first reported study of obstetric and neonatal interprofessional learning acquired via an online simulation-based team training workshop in India. Our results show that key characteristics of crisis resource management like teamwork, communication, leadership, using all available resources, continuous reassessment and avoidance of fixation of ideas [35] can be successfully acquired via an online format. We have reported the use of a Community of Inquiry framework for conducting online training workshops. The format and framework of CoI can possibly be transferrable to other team training workshops in LMICs as well as in well-resourced countries. The study is timely and relevant given the need for physical distancing because of the pandemic and future outbreaks.

The online workshop relies on availability of a good internet service and digital infrastructure that may not be easily available in all LMICs. This could be a limitation for online delivery of SBE in general. In-person training cannot be fully replaced by online workshops since this does not give students the opportunity to acquire skills-based training (for e.g. hands-on learning the manoeuvres for management of shoulder dystocia). Further studies are needed to ascertain if our results can be replicated in other LMICs and in other specialities like emergency medicine. Further inductive analysis would provide useful insights into the full impact of CoI in SBE. We plan to further analyse and present our findings in a future publication.

## Conclusions

Community of Inquiry framework serves as an effective lens to describe online education. The results of our study show that the online format is a feasible model for continuing to provide SBE while adhering to distancing protocols. The CoI framework aligns well with the unique demands of online simulation, and further studies using this model could inform future online education programmes. Our study shows that it is possible to build a community of inquiry using online simulation. Furthermore, the learnings from in-person simulation can be replicated using online SBE.

This is the first reported study in literature that evaluates online interprofessional, simulation-based workshop for management of perinatal emergencies in an LMIC setting. More studies, using robust frameworks such as CoI, may be helpful to evaluate the role of online simulation in learning.

## Data Availability

Raw data is available from authors on reasonable request.

## Abbreviations

CoI: Community of Inquiry
HIMS: Hind Institute of Medical Sciences
IPE: Interprofessional education
LMIC: Low- and middle-income country
ONE-Sim: Obstetric neonatal emergency simulation
PPE: Personal protective equipment
SBE: Simulation-based education
